# CD8^+^ T Cells in OA Knee Joints Are Differentiated into Subsets Depending on OA Stage and Compartment

**DOI:** 10.3390/jcm11102814

**Published:** 2022-05-17

**Authors:** Hadrian Platzer, Richard Trauth, Timo A. Nees, Elena Tripel, Simone Gantz, Marcus Schiltenwolf, Babak Moradi, Nils Rosshirt

**Affiliations:** 1 Clinic for Orthopedic and Trauma Surgery, University Hospital Heidelberg, 69118 Heidelberg, Germany; hadrian.platzer@med.uni-heidelberg.de (H.P.); richard.trauth@yahoo.de (R.T.); timo.nees@med.uni-heidelberg.de (T.A.N.); elena.tripel@med.uni-heidelberg.de (E.T.); simone.gantz@med.uni-heidelberg.de (S.G.); marcus.schiltenwolf@med-heidelberg.de (M.S.); 2 Clinic for Orthopedics and Trauma Surgery, University Hospital Kiel, 24105 Kiel, Germany

**Keywords:** osteoarthritis, T cells, CD8, synovial membrane, inflammation, flow cytometry

## Abstract

Osteoarthritis (OA) is no longer considered a purely degenerative disease. OA is defined as a disease of the entire joint, in which inflammation occurs in various joint tissues. The overall aim of this study was to analyze the presence and polarization of CD8^+^ T cell subsets in OA knee joints, in relation to the OA stage and compartment (synovial fluid (SF), synovial membrane (SM,) peripheral blood (PB)). A quantitative flow analysis of CD8^+^ T cell subsets to compare the SF, SM, PB, was performed in patients with different stages of OA (early, unicondylar and bicondylar OA). Samples of the SF, SM and PB were harvested from a total of 55 patients at the time of surgery. Early OA was confirmed by independent surgeons intraoperatively. Uni- and bicondylar OA was confirmed and graded by two plane radiographs. Samples were analyzed by flow cytometry for surface markers, and cytokines by intracellular staining (ICS). CD8^+^ T cells were shown to be differentiated into pro-inflammatory IFN-γ producing Tc1 and IL-17A producing Tc17, as well as anti-inflammatory IL-4 producing Tc2. All CD8^+^ T cell subsets (Tc1, Tc17, and Tc2) were detected in both the SM and SF. The percentage of CD8^+^ T cell subsets of the total CD8^+^ T cell population was dependent on the OA stage and compartment. Compared with the peripheral blood (PB), the proportion of CD8^+^IFN-γ^+^ Tc1 and CD8^+^IL-17A^+^ Tc17 was significantly increased in OA SF. This was confirmed in our data for both early OA and end-stage OA. In the SM samples of end-stage OA patients, the proportion of CD8^+^IL-17A^+^ Tc17 was significantly increased compared to the PB. Comparing SF and SM samples of end-stage OA patients, the proportion of CD8^+^IFN-γ^+^ Tc1 was significantly increased in SF, whereas there were no differences concerning CD8^+^IL-4^+^ Tc2 and CD8^+^IL-17A^+^ Tc17. End-stage OA samples showed a significant increase of CD8^+^IL-4^+^ Tc2 in the SM for both unicondylar and bicondylar OA compared to early OA. CD8^+^ T cells infiltrating the SM and SF in OA knees are differentiated into IFN-γ-, IL-17A-, and IL-4-producing CD8^+^ T cell subsets (Tc1, Tc17, Tc2). This differentiation depends on the OA stage and OA compartment. Further investigation of CD8^+^ T cell subsets and their interaction with other inflammatory cells such as CD4^+^ T cells and macrophages may help to identify novel therapeutic anti-inflammatory strategies for containing OA progression.

## 1. Introduction

Osteoarthritis (OA) is considered the most prevalent joint disease [1], but therapeutic strategies have neither changed nor improved significantly in recent decades. While joint arthroplasty is often a sufficient therapeutic option for end-stage OA, therapeutic strategies for early OA solely focus on symptom reduction, and are often inadequate and have no impact on disease pathology and progression [2,3,4]. Considering that OA is not only a degenerative disease and inflammation takes place in various joint tissues, especially synovial tissue, leading to cartilage destruction and disease progression [5,6,7,8], the perspective of developing new anti-inflammatory therapeutic strategies to halt disease progression should come into focus.

Previous studies have shown that macrophages and T cells particularly infiltrate OA joints [8], resulting in increased levels of proinflammatory cytokines and metalloproteinases [5,9].

These studies focused mainly on macrophages and CD4^+^ T cells. Nevertheless, various studies have demonstrated the presence of CD8^+^ T cells in synovial tissue and the synovial fluid of OA joints [10,11,12,13]. Further, several studies indicated an involvement of CD8^+^ T cells in OA disease [14,15] and, by utilizing a post-traumatic OA mouse model, it was shown that synovial CD8^+^ T cells exhibit an active phenotype once OA pathology is induced [14]. However, it is still controversial how much CD8^+^ T cells in the synovial fluid (SF) and synovial membrane (SM) really contribute to OA pathology [16,17]. The transfer from detection of CD8^+^ T cells in the SF and SM to identifying the actual role in OA pathology continues to be the missing link, especially since differentiation and, thereby, phenotype of CD8^+^ T cells in OA has not yet been sufficiently investigated.

There is increasing evidence that CD8^+^ T cells, like CD4^+^ T cells, can be divided into different subsets that secrete specific effector cytokines. The previous literature has shown that CD8^+^ T cells can be differentiated into IFN-γ-producing Tc1, IL-4-producing Tc2, and IL17-producing Tc17 [18,19], but whether this differentiation is taking place in OA joints has not yet been sufficiently analyzed.

The main objective of this study was to investigate whether CD8^+^ T cell subsets are present in OA knee joints. In addition, we aimed to analyze whether differentiation of CD8^+^ T cell into subsets differs between compartments (SF and SM) or if it depends on OA stage.

## 2. Materials and Methods

### 2.1. Study Population

A total of 55 patients (32 women, 23 men) were enrolled in this study. Of those, 14 presented with arthroscopic or MRI findings of early osteoarthritis of the knee and Kellgren–Lawrence grade 0-I on two-plane radiographs, and were scheduled for arthroscopic surgery at our hospital. Forty-one patients were diagnosed with end-stage OA by two plane radiographs. Of those, 19 had unicondylar OA and 22 had bicondylar OA and were scheduled for arthroplastic knee surgery. No patient of the early OA group underwent surgery directly after trauma. The minimum time after trauma/onset of symptoms was 6 weeks, the median was 9 months (Table S1). The International Cartilage Repair Society (ICRS) grade was assessed by independent surgeons intraoperatively. All patients included had an ICRS grade I-IV in at least two compartments (5%), or grade II-IV in one compartment (95%), and met the criteria of early OA as previously suggested by Luyten et al. [20,21]. For ethical reasons, samples from healthy donors were not included in this study. None of the patients had a history of underlying inflammatory disease, intake of a DMARD, intra-articular injection of corticosteroids, hyaluronic acid or regular intake of an NSAID. Systemic inflammatory parameters (C-reactive protein (CRP) and white blood cells (WBC)) were within the physiological range at the time of surgery (Table 1). The mean age of the study population was 60.6 ± 15.2 years. The underlying pathologies for arthroscopic knee surgery were meniscal tear (35.7%), traumatic anterior cruciate ligament (ACL) tear (14.3%), and localized cartilage damage (50%) (Table S1). The ethics committee of the University of Heidelberg approved this study (S-333/2007). Written informed consent from all patients was obtained prior to study enrollment.

### 2.2. Sample Collection

Synovial fluid (SF), synovial membrane (SM) and peripheral blood (PB) were harvested at the time of surgery. SF was aspirated prior to the establishment of the arthroscopic portals, respectively, prior to arthrotomy into sterile tubes, and further processed as described below. SM biopsies were performed intra-operatively from the suprapatellar pouch as previously described [22]. EDTA-anticoagulated PB samples were taken concurrently at the time of surgery.

### 2.3. Cell Preparation and Isolation

SF samples were treated with bovine testicular hyaluronidase (1 mg/mL, Sigma-Aldrich, St. Louis, MO, USA) for 30 min at 37 °C and washed twice with phosphate buffered saline (PBS). SM samples were rinsed twice with PBS, minced finely with sterilized scissors and digested with collagenase B (1 mg/mL; Roche, Indianapolis, IN, USA) and bovine testicular hyaluronidase IV (2 mg/mL; Sigma-Aldrich, USA) at 37 °C for 2 h in RPMI 1640 culture medium (Invitrogen, Waltham, MA, USA) supplemented with 10 μg/mL penicillin–streptomycin (Invitrogen, USA) and 10% FCS (Biochrom AG, Berlin, Germany). The cell suspension was filtered through a 100 μm (BD Biosciences, San Jose, CA, USA) and a 40 μm pore-size cell strainer (EMD Millipore, Burlington, MA, USA) to remove any undigested tissue. The filtered cell suspension was washed twice with PBS. Mononuclear cells were isolated from anticoagulated PB and SM cell suspensions using Ficoll-Paque TM PLUS (GE Healthcare, Chicago, IL, USA) density gradient centrifugation. T cells were isolated from PB and SM mononuclear cells by CD3 MACS bead separation (Miltenyi Biotec, Bergisch Gladbach, Germany).

### 2.4. Flow Cytometry Analysis of Cell Surface Markers and Intracellular Staining

Multi-color flow cytometry was used to identify CD8^+^ T cell subsets by their preferential expression of intracellular markers. In brief, for the staining of intracellular markers, MACS CD3 isolated T cells from the PB and SM, and cells from the SF were taken into culture at a final density of 10^6^ cells/mL and incubated for 10 h at 37 °C/5% CO_2_. Cell cultures were stimulated with phorbol myristate acetate (PMA) (50 ng/mL) and ionomycin (1 μg/mL). After 4 h of activation, brefeldin A (5 μg/mL, Sigma-Aldrich, Germany) was added for another 2 h. After a total of 6 h of activation, cells were collected, washed twice in FACS buffer, and blocked with FCS blocking reagent. For surface marker expression VioBlue-labelled mAb against CD8 (clone BW135/80, Miltenyi Biotec, Germany) was used and staining was performed at 4 °C for 30 min. Following surface staining, cells were fixed and permeabilized using a cytofix/cytoperm reagent and then stained with APC (Allophycocyanin) labelled anti-IFN-γ (antibody against interferon-gamma; clone B27), FITC (Fluorescein isothiocyanate) labelled anti–IL-4 (antibody against interleukin-4; clone MP4-25D2) and PE (Phycoerythrin) labelled anti-IL-17A (antibody against interleukin-17A; clone N49-653). Dead cells were not excluded in the flow analysis, which is a limitation of this study. Cytokine values for CD8^+^ T cells were measured by flow cytometry.

**Figure 1:**
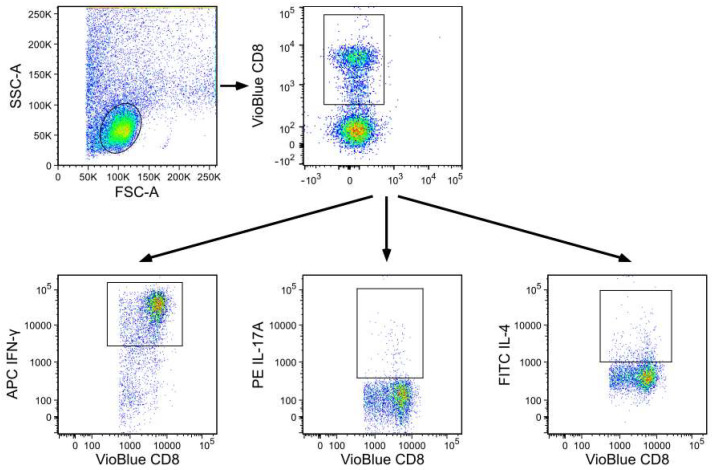
Flow cytometric gating strategy. The gating strategy for analysis of CD8^+^ T cell subsets is presented by a representative flow cytometry analysis of an SF sample from a patient with unicondylar knee OA. First, cells were gated based on their forward-/side-scatter (FSC/SSC) profile. Cell debris was excluded. Then CD8^+^ T cell subsets were analyzed for a CD8 surface marker and for intracellular staining of IFN-γ, IL-17A and IL-4.

Flow analysis was performed using a MACSQuant Analyzer (Miltenyi Biotec, Germany), which is a seven-channel flow cytometer. Data analysis was performed using FlowJo v10.8.1 (BD Biosciences, USA). The gating strategy is presented in Figure 1. The cut-off for all cell surface markers was defined based on autofluorescence controls. Antibodies and cell preparation solutions were purchased from BD Biosciences, USA, if not stated otherwise.

### 2.5. Statistical Analysis

Data are presented as the arithmetic mean ± standard deviation, if not stated otherwise. Gaussian distribution was assessed by the Kolmogorov–Smirnov test. To compare concurrent SF, SM and PB samples, we used the Kruskal–Wallis test for non-parametric data with Mann–Whitney U post hoc test and Bonferroni correction. For correlation analyses, the Pearson Correlation coefficient was utilized. *p* values ≤ 0.05 were considered significant. Statistical analysis was performed using IBM SPSS Statistics v27 (IBM, Armonk, NY, USA).

## 3. Results

To investigate CD8^+^ T cell subsets in OA joints depending on OA stage and compartment, samples of SF, SM, and PB were analyzed from patients with early OA and end-stage unicondylar and bicondylar OA by flow cytometry. IFN-γ, IL-17A and IL-4 producing CD8^+^ T cell subsets (Tc1, Tc17, Tc2), were detected and quantified.

### 3.1. Differentiation of CD8^+^ T Cell Subsets in the SF Compared to PB

**Figure 2:**
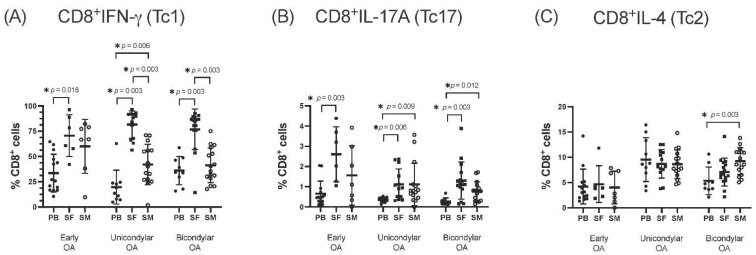
Comparison of CD8^+^ T cell subsets depending on Osteoarthritis (OA) stage. Frequency of CD8^+^ T cell subsets Tc1, Tc17, and Tc2, in relation to the proportion of the total CD8^+^ T cell population, are presented, depending on the OA stage. In short, multi-color flow cytometry was used to identify CD8^+^ T cell subsets in the peripheral blood (PB), synovial fluid (SF) and synovial membrane (SM) by their preferential expression of extracellular and intracellular markers. Significant differences are indicated by asterisks: * *p* ≤ 0.05.

In both, early- and end-stage OA, the percentages of CD8^+^IFN-*γ*^+^ (Tc1) and CD8^+^IL-17A^+^ (Tc17) of the total CD8^+^ T cell population were significantly increased in the SF compared with PB (Figure 2A,B). No significant differences were observed between the SF and PB when comparing the percentage of CD8^+^IL-4^+^ Tc2 (Figure 2C).

### 3.2. Differentiation of CD8^+^ T Cell Subsets in SM Compared to PB

Early OA samples showed no significant differences in the analysis of CD8^+^ T cell subsets of the SM compared to PB. End-stage OA samples, however, revealed a significantly increased proportion of CD8^+^IL-17A^+^ (Tc17) in the SM compared with PB, for both unicondylar and bicondylar OA (Figure 2B). Further, the proportion of CD8^+^IFN-*γ*^+^ (Tc1) in the SM was significantly increased with 42.0 ± 20.0% in unicondylar OA compared to 19.7 ± 16.8% in PB. No significant differences were observed for bicondylar OA compared with PB in this regard (Figure 2A). In the SM of bicondylar OA, but not unicondylar OA, the proportion of CD8^+^IL-4^+^ (Tc2) was significantly increased compared to PB (Figure 2C).

### 3.3. Comparison of CD8^+^ T Cell Differentiation between Joint Compartments—The SF versus the SM

The proportion of CD8^+^IFN-*γ*^+^ (Tc1) was significantly increased in the SF for both end-stage OA groups with 81.6 ± 13.5% in unicondylar OA and with 76.9 ± 20.1% in bicondylar OA compared to the SM (42.0 ± 20.0% and 41.3 ± 17.2%, respectively) (Figure 2A). No significant differences for CD8^+^ T cell subset differentiation were detected between the SF and SM in samples with early OA.

### 3.4. Differentiation of CD8^+^ T Cell Subsets in Relation to OA Stage

**Figure 3:**
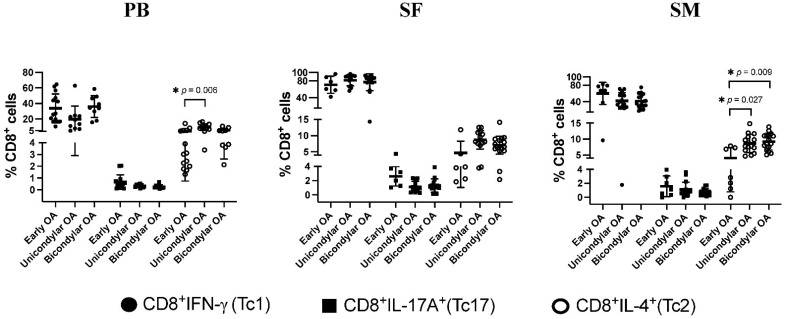
Comparison of CD8^+^ T cell subsets depending on joint compartment. Percentage rates of CD8^+^ T cell subsets Tc1, Tc17, and Tc2, and their proportion of the total CD8^+^ T cell population are presented, depending on the joint compartment. In short, multi-color flow cytometry was used to identify CD8^+^ T cell subsets in the peripheral blood (PB), synovial fluid (SF) and synovial membrane (SM) by their preferential expression of extracellular and intracellular markers. Significant differences are indicated by asterisks: * *p* ≤ 0.05.

When OA stages were compared depending on the compartment (SF, SM, and PB), no significant differences were observed between early, uni- and bicondylar OA regarding the infiltration of IFN-γ and IL-17A producing CD8^+^ Tc1 and Tc17 (Figure 3). In SM samples though, the proportion of CD8^+^IL-4^+^ (Tc2) in end-stage OA was significantly increased for both uni-, and bicondylar with 8.6 ± 2.9% in unicondylar OA and 9.2 ± 2.6% in bicondylar OA, compared to 3.8 ± 3.1% in early OA (Figure 3).

### 3.5. Tc1/Tc2 and Tc17/Tc2 Relation Dependent on K&L Score

Our data revealed a low to moderate negative correlation varying from −0.290 to −0.504 between pro-inflammatory CD8^+^ T cell differentiation and OA grade for both the SF and SM, meaning that pro-inflammatory polarization defined by the ratio of Tc17/Tc2 and Tc1/Tc2 decreased with a higher K&L score. A significant correlation was observed for Tc17/Tc2 for both the SF and SM (*p* = 0.002 *), while the correlation of Tc1/Tc2 and K&L grade was only significant for the SM (*p* = 0.008 *) (Table 2).

## 4. Discussion

Inflammation in OA joint tissues is considered to be a critical factor in OA progression. It was shown that infiltration of macrophages and CD4^+^ T cell subsets contributes to increased levels of inflammatory cytokines and chondro-destructive enzymes in OA joints [5,9], leading to an imbalance between anabolic and catabolic processes and thus resulting in cartilage degeneration. Although the presence of CD8^+^ T cells in OA joints has already been demonstrated [10,11,12,13], analyses of CD8^+^ T cell subsets are lacking. Recently, it has been shown that, not only CD4^+^ T cells, but also CD8^+^ T cells, can be classified into different subsets characterized by their effector cytokines [18,19]. The overall aim of this study was to analyze CD8^+^ T cell subsets depending on the OA stage and compartment. To our knowledge, this is the first study analyzing the polarization status of CD8^+^ T cell subsets in patients with knee OA. Because the age of included patients in this study differs between early, unicondylar and bicondylar OA groups, justified by the age-related frequency of included diseases and surgeries, age could be considered as a confounding factor in this study. Further, as found out recently for SF samples from OA patients, gender could also be a confounding factor [23]. We thoroughly controlled for gender-related influences but found no differences (data not shown). Therefore, varying gender ratios between the groups do not seem to affect the statistical validity of our data. In this study, pro-inflammatory IFN-γ (Tc1) and IL-17A (Tc17), as well as anti-inflammatory IL-4 (Tc2) producing CD8^+^ subsets were shown to be present in the SF and the SM of patients with knee OA. Their proportion of the total CD8^+^ T cell population was dependent on the stage and compartment of OA. While IL-4 is known for its anti-inflammatory effects in OA [24], IFN-γ and IL17A are potent and biologically active pro-inflammatory cytokines that, in general, promote inflammation. IL-17A is known for its inhibition of proteoglycan synthesis by chondrocytes [25]. Further, IL17A was shown to induce collagenase-3 production in chondrocytes [26] and to stimulate the release of chemokines by chondrocytes and synovial fibroblasts [27], thereby impairing cartilage homeostasis. IFN-γ induces apoptosis in chondrocytes and is a well-known activator of macrophages [28,29]. Due to the low cell number, CD3 MACS isolation was not performed for the SF samples, so the SF results may have been influenced by the presence of other CD8^+^ cells, such as a subset of natural killer cells, which is a limitation of this study.

Compared with PB, the proportion of pro-inflammatory CD8^+^ T-cell subsets Tc1 and Tc17 of the total CD8^+^ T cell population was significantly increased in the SF of patients with knee OA, independent of OA stage. In the SM, pro-inflammatory polarization was only found for unicondylar OA with a significantly increased proportion of Tc1 and Tc17 compared with the PB. No significant differences were found for the anti-inflammatory IL-4 producing CD8^+^ Tc2 subset between the SM and PB in this regard. Pro-inflammatory polarization of CD8^+^ T cells in OA joints is apparently more present in earlier stages of the disease, as the Tc1/Tc2 and Tc17/Tc2 ratio showed a moderately negative correlation to K&L grade in the SF and SM, supporting the results previously presented by our group and others [6,28,29]. Considering the pro-inflammatory effects of IFN-γ and IL17A, the results from this study show that CD8^+^ T cells could significantly contribute to inflammatory burden in OA knee joints, especially during disease onset. The shift from Tc2 towards the pro-inflammatory Tc1 and Tc17 subsets, predominantly in patients with low K&L scores, indicates a higher inflammatory impact of CD8^+^ T cells in OA onset, making CD8^+^ T cell subsets a potential target for an anti-inflammatory, chondroprotective therapy.

Inflammatory markers have previously been shown to be elevated in the peripheral blood of OA patients compared with healthy controls [13,30]. In this study, The CD8^+^ Tc2 subset was reduced in the PB of patients with early OA compared with the PB of patients with unicondylar OA, which may reflect a measurable systemic radiation of locally uncontrolled inflammation in OA pathophysiology.

It has previously been shown that OA progression is characterized by a distinct influx of inflammatory cells [31]. The results of this study confirm this finding. It is not yet known whether unicondylar and bicondylar knee OA are two different stages of the same disease or even different subtypes of knee OA with a different underlying etiology.

In a previous study we demonstrated that the inflammatory pattern differs between unicondylar and bicondylar knee OA with a significantly higher cytokine concentration in the SF and significantly higher frequencies of CD14^+^ macrophages, CD4^+^ and CD8^+^ T cells in the SM of patients with bicondylar OA [32]. If unicondylar and bicondylar OA represent two different types of OA, the results of this study demonstrate that the polarization of CD8^+^ T cells toward both pro-inflammatory Tc1 and Tc17 subsets in the SM is a specific inflammatory feature of unicondylar OA. In this study, the SM samples of bicondylar OA, but not from unicondylar OA, showed a significantly increased proportion of the CD8^+^ Tc2 subset compared to the PB, indicating a stronger anti-inflammatory impact of CD8^+^ T cells in the SM from patients with bicondylar OA compared to unicondylar OA.

If the theory of different stages (early knee OA; unicondylar OA; bicondylar OA) is adopted, these OA stages could each be defined by a different inflammatory pattern in terms of infiltration and polarization of CD8^+^ T-cell subsets. Early OA could be characterized by a significant pro-inflammatory polarization of CD8^+^ T cells toward Tc1 and Tc17 subsets in the SF, while lacking significant differences in the SM. Why there is such a big difference between the SF and SM remains unclear. It is conceivable, that local chemokine and cytokine, as well as adhesion molecule expression, effectuates influx of already differentiated pro-inflammatory CD8^+^ T cell subsets from the sub-lining of the SM layer into the synovial fluid, as previously described [11,33,34]. Progression of OA toward unicondylar OA could be characterized by the pro-inflammatory polarization of CD8^+^ T cell subsets in the SF and additionally in the SM. This pro-inflammatory polarization of CD8^+^ T cells in the SM could thus be an indicator of progression of OA from early to unicondylar OA. A significantly increased proportion of Tc2 in the SM could then in turn be an indicator of progression from uni- to bicondylar OA. Choosing the optimal surgical therapy for unicondylar and bicondylar OA is sometimes challenging. Currently, the combination of patient symptoms, knee examination, and radiographic analysis determine the surgical procedure. Nevertheless, the boundaries between unicondylar and bicondylar knee OA are blurred, and often the decision to perform an unicondylar or bicondylar arthroplastic surgery is difficult. The results of this study indicate that, in addition to considering patient symptoms and radiographic data, analysis and comparison of CD8^+^ T cell subsets in both the SM and PB before surgery could help to better define the OA phenotype to choose the optimal surgical therapy.

## 5. Conclusions

Different subsets of inflammatory cells are present in OA joints and contribute to increased levels of chondro-destructive enzymes and cytokines. This study shows that CD8^+^ T cells in OA knee joints can be classified into Tc1, Tc2 and Tc17 subsets based on their cytokine production. Further, the data from this study show that, in knee OA, these CD8^+^ T cell subsets are polarized toward inflammatory phenotypes Tc1 and Tc17 in the SF compared to the PB, revealing that CD8^+^ T cells may contribute substantially to the inflammatory burden in OA, which in turn is associated with increased cartilage destruction and disease progression.

The results of this study indicate that CD8^+^ T cell subsets may be targets of anti-inflammatory strategies in OA. Therefore, further studies are needed to investigate CD8^+^ T cell subsets and their interaction with surrounding cells such as fibroblasts, CD4^+^ T cells, and macrophages.

## Figures and Tables

**Table 1:** Characteristics of the study population.

	Total Study Population	Early OA	Unicondylar OA	Bicondylar OA
Number of patients, *n*	55	14	19	22
Gender, *n* (%)				
Male	23 (41.8%)	4 (28.6%)	11 (58.0%)	8 (36.4%)
Female	32 (58.2%)	10 (71.4%)	8 (42.1%)	14 (63.6%)
Age at surgery, years	60.6 ± 15.2	41.9 ± 13.1	65.0 ± 9.6	68.8 ± 9.3
Mean ± s.d. (IQR)	(52.5–72)	(31.3–47.8)	(60.5–69.5)	(61.3–74.8)
BMI, kg/m^2^	29.1 ± 5.8	25.6 ± 3.6	29.9 ± 5.5	30.6 ± 6.4
Mean ± s.d. (IQR)	(25.2–32.4)	(22.6–28.1)	(26.9- 32.8)	(25.6–34.8)
Leukocyte cells/nl	7.0 ± 1.8	6.6 ± 1.7	7.1 ± 1.3	7.1 ± 2.2
Mean ± s.d. (IQR)	(5.9–7.6)	(5.5–7.5)	(6.4–7.5)	(5.8–8.6)
C-reactive protein, mg/L	3.7 ± 2.5	3.4 ± 2.0	3.9 ± 2.4	3.8 ± 3.0
Mean ± s.d. (IQR)	(2.0–4.4)	(2.0–4.0)	(2.0–5.7)	(2.0–4.4)
K&L Score, *n* (%)				
1	9 (16%)	9 (64%)		
2	5 (9%)	5 (36%)		
3	29 (53%)		19 (100%)	10 (45%)
4	12 (22%)			12 (55%)

Demographic and clinical parameters of the study population are displayed. Data are presented as mean ± standard deviation (range) or as number (p%). IQR = interquartile range, BMI = body mass index, K&L Score = Kellgren and Lawrence score. OA = Osteoarthritis

**Table 2:** Correlation analysis of inflammatory polarization of CD8^+^ T cell subsets and Osteoarthritis (OA) grade.

	Pearson-Correlation Coefficient	
	(CD8^+^IFN-γ^+^/CD8^+^IL-4^+^)/K&L Score	(CD8^+^IL-17A^+^/CD8^+^IL-4^+^)/K&L Score
**SF**	−0.290	−0.504
*p*-value	0.091	0.002 *
**SM**	−0.429	−0.497
*p*-value	0.008 *	0.002 *

Pearson correlation coefficients are shown. In brief, inflammatory polarization of CD8^+^ T cell subsets, defined as ratio of pro-inflammatory Tc1 (CD8^+^IFN-*γ*^+^) or Tc17 (CD8^+^IL17A^+^) and anti-inflammatory Tc2 (CD8^+^IL-4^+^) was correlated with OA grade defined by K&L score (Kellgren and Lawrence score). Significant correlation coefficients are indicated by asterisks: * *p* ≤ 0.05.

## Supplementary Materials

The following supporting information can be downloaded at: https://www.mdpi.com/article/10.3390/jcm11102814/s1, Table S1: Clinical features of the early OA group; Figure S1: Correlation analysis of inflammatory polarization of CD8^+^ T cell subsets and OA grade; Figure S2: Comparison of CD8^+^ T cell subset MFI values for uni- and bicondylar OA.
