# Supplementary material for: CD8+ T Cells in OA Knee Joints Are Differentiated into Subsets Depending on OA Stage and Compartment
Source: J Clin Med. 2022 May 17;11(10):2814. doi: 10.3390/jcm11102814 (PMC9145354; doi:10.3390/jcm11102814)
Supplement: Supplementary file 1 [file jcm-11-02814-s001.zip › jcm-1661785-Supplementary.pdf]

Supplementary Table S1. Clinical features of the early OA group.

**Time from onset of symptoms/trauma to operation in months, *n (%)***		
1-3		2 (14.3 %)
4-6		4 (28.5 %)
7-9		3 (21.4 %)
10-12		2 (14.3 %)
>12		3 (21.4 %)
**ICRS grade**		
I-IV in ≥ 2 compartments		2 (14.3 %)
II-IV in 1 compartment		12 (85.7 %)
**Pathology, *n (%)***		
Meniscus tear		5/14 (35.7 %)
ACL tear		2/14 (14.3 %)
Localized cartilage damage		7/14 (50 %)

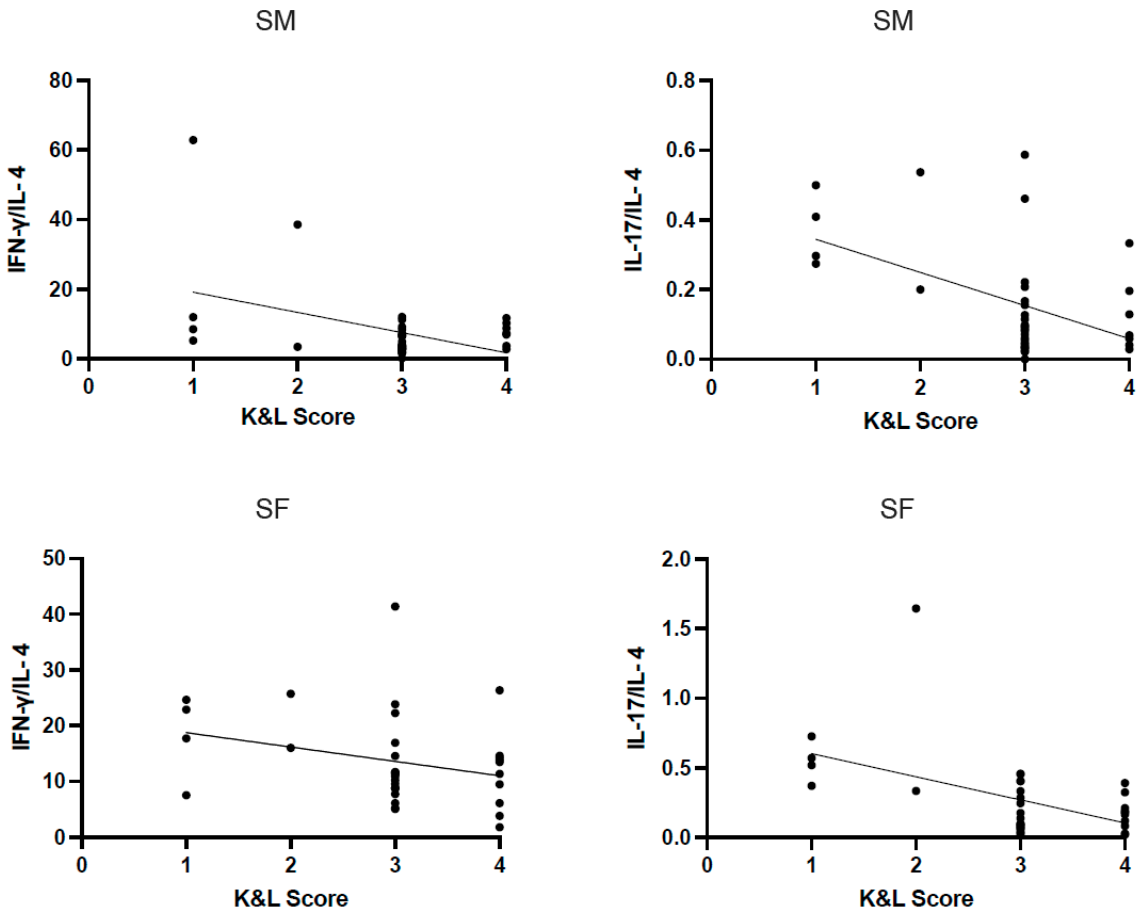

Supplementary Figure S1: Correlation analysis of inflammatory polarization of CD8⁺ T cell subsets and OA grade.

Correlation plots of (CD8⁺IFN-γ⁺/CD8⁺IL-4⁺) / K&L Score and (CD8⁺IL-17A⁺/CD8+IL-4⁺) / K&L Score and linear regression lines are shown.

In brief in samples of synovial fluid (SF) and synovial membrane (SM), inflammatory polarization of CD8⁺ T cell subsets, defined as ratio of pro-inflammatory Tc1 (CD8⁺IFN-γ⁺) or Tc17 (CD8⁺IL17A⁺) and anti-inflammatory Tc2 (CD8⁺IL-4⁺) was correlated with OA grade defined by K&L score (Kellgren and Lawrence score).

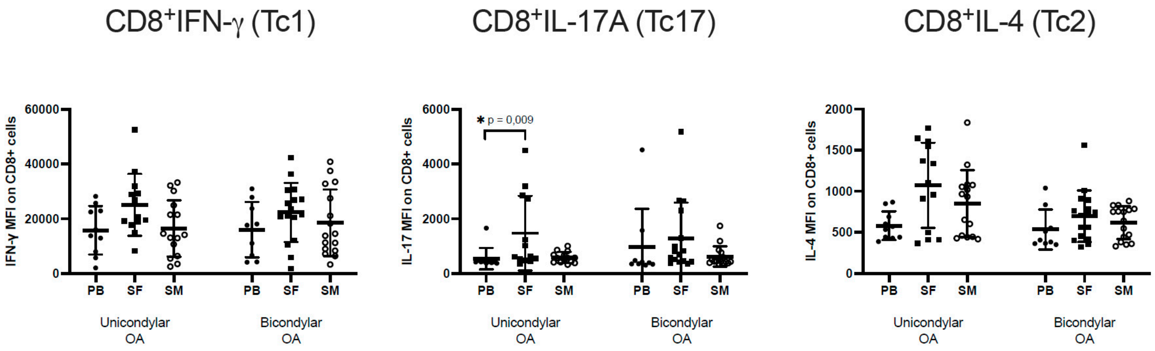

Supplementary Figure S2: Comparison of CD8⁺ T cell subset MFI values for uni- and bicondylar OA. MFI values of CD8⁺ T cell subsets Tc1, Tc17 and Tc2 are presented for uni- and bicompartmental OA, depending on joint compartment. In short, multi-color flow cytometry was used to identify CD8+ T cell subsets in peripheral blood (PB), synovial fluid (SF) and synovial membrane (SM) by their preferential expression of extracellular and intracellular markers. Significant differences are indicated by asterisks: *$p \leq 0.05$.
